# Electrocardiographic effect of artemisinin-piperaquine, dihydroartemisinin-piperaquine, and artemether-lumefantrine treatment in falciparum malaria patients

**DOI:** 10.1590/0037-8682-0536-2020

**Published:** 2021-02-10

**Authors:** Wanting Wu, Chenguang Lu, Yuan Liang, Hongying Zhang, Changsheng Deng, Qi Wang, Qin Xu, Bo Tan, Chongjun Zhou, Jianping Song

**Affiliations:** 1 Guangzhou University of Chinese Medicine, Artemisinin Research Center, Guangzhou, Guangdong, People’s Republic of China.; 2 Guangzhou University of Chinese Medicine, Sci-tech Industrial Park, Guanzhou, Guangdong, People’s Republic of China.; 3 Guangzhou University of Chinese Medicine, Institute of Tropical Medicine, Guangzhou, Guangdong, People’s Republic of China.

**Keywords:** QT prolongation, ACT, Antimalarial, ECG

## Abstract

**INTRODUCTION:**

Artemisinin-based combination therapy (ACT), such as artemisinin-piperaquine (AP), dihydroartemisinin-piperaquine (DP), and artemether-lumefantrine (AL), is the first-line treatment for malaria in many malaria-endemic areas. However, we lack a detailed evaluation of the cardiotoxicity of these ACTs. This study aimed to analyze the electrocardiographic effects of these three ACTs in malaria patients.

**METHODS:**

We analyzed the clinical data of 89 hospitalized patients with falciparum malaria who had received oral doses of three different ACTs. According to the ACTs administered, these patients were divided into three treatment groups: 27 treated with AP (Artequick), 31 with DP (Artekin), and 31 with AL (Coartem). Electrocardiograms and other indicators were recorded before and after the treatment. The QT interval was calculated using Fridericia’s formula (QTcF) and Bazett’s formula (QTcB).

**RESULTS:**

Both QTcF and QTcB interval prolongation occurred in all three groups. The incidence of such prolongation between the three groups was not significantly different. The incidence of both moderate and severe prolongation was not significantly different between the three groups. The ΔQTcF and ΔQTcB of the three groups were not significantly different. The intra-group comparison showed significant prolongation of QTcF after AL treatment.

**CONCLUSIONS:**

Clinically recommended doses of DP, AL, and AP may cause QT prolongation in some malaria patients but do not cause torsades de pointes ventricular tachycardia or other arrhythmias.

## INTRODUCTION

According to the World Malaria Report published by the World Health Organization (WHO) in 2019, the global incidence rate of malaria has decreased from 71 cases per 1000 people in 2010 to 57 cases per 1000 people in 2014, but this rate of decline has begun to slow down since 2014^1^. Malaria is a disease that is prevalent in poor regions or countries. The costs of prevention, diagnosis, and treatment are very burdensome for these regions and countries^2^. The WHO recommends artemisinin-based combination therapy (ACT) for malaria. ACT means a combination of an artemisinin derivative with a longer-acting antimalarial that has a different mode of action. High cost and drug resistance have become global problems^3^. Therefore, effective and low-cost drugs are of great significance. 

ACT has been used as the first-line treatment for uncomplicated *Plasmodium falciparum* malaria in more than 80 countries^4^, with more than 10 years of such use in many of these countries^5^ ^,^ ^6^. Using ACT results in rapid elimination of asexual parasitemia^7^ ^,^ ^8^ and alters the sex ratio of gametophytes, thus reducing the infectivity of malaria^9^. A study proposed that ACT treatment can transfer immature gametocytes from the bone marrow to the peripheral blood and rapidly clear them^9^. Artemisinin and its derivatives work rapidly, but because of their short half-lives^10^ ^,^ ^11^, the monotherapy courses for vivax and falciparum malaria are 5 and 7 days, respectively. Such a long duration of the course makes it difficult to use these drugs widely in malaria-endemic regions, such as Southeast Asia and Africa. Therefore, the course of treatment was shortened to 3 days with a combination therapy^12^ ^-^ ^15^.

The WHO redefined mass drug administration (MDA) in 2017^16^. MDA is the administration of a full therapeutic course of antimalarial medicine to every person in specific areas, including infected and uninfected people, except to those with drug contraindications. The individuals are administered these drugs at approximately the same time and often at repetitive intervals. The objective of MDA is to administer therapeutic doses of antimalarials to a large proportion of a specific population to eliminate symptomatic and asymptomatic infections, prevent reinfection, and contain the spread of malaria. The participating MDA population includes malaria-infected individuals and many healthy individuals; hence, the ideal antimalarial drug must be safe and long-acting to prevent reinfection and relapse^17^. Studies have reported that MDA with artemisinin-piperaquine (AP) effectively reduced the prevalence of malaria without causing serious adverse reactions^18^ ^,^ ^19^.

With reports of many antimalarial drugs involving QT prolongation, the cardiotoxicity of such drugs has received renewed attention. Prolongation of ventricular repolarization can lead to effective refractory period prolongation, and an electrocardiogram (ECG) will reflect the QT interval prolongation^20^. The QT interval varies with the heart rate; hence, the corrected QT (QTc) interval should be used to evaluate the cardiac effects of these drugs. Drug-induced QT/QTc interval prolongation may increase the risk of torsades de pointes ventricular tachycardia (TdP), owing to which cardiotoxicity of antimalarial medicines has regained attention^21^ ^,^ ^22^. TdP is a type of polymorphic ventricular arrhythmia related to QT interval prolongation. A prolonged QT interval followed by rapid and disorganized contraction of the heart leads to TdP. Transient TdP can cause clinical symptoms, such as dizziness or loss of consciousness, and even death in severe cases. Though not all QT prolongations cause TdP, they are common biomarkers for identifying drugs that may cause it^23^. A study confirmed the effects of AP compounds on the ECG of malaria patients; it found that AP compounds caused QT interval prolongation in some malaria patients but without TdP^24^. The aim of this study was to compare the effects of dihydroartemisinin-piperaquine (DP), artemether-lumefantrine (AL), and AP on the ECGs of malaria patients.

## METHODS

We conducted a retrospective analysis of clinical data of 89 hospitalized falciparum malaria patients who had received oral doses of three different ACTs in Cambodia in 2005. Positive results for rapid diagnostic tests (RDTs) and microscopy were used as the basis for laboratory-verified cases. Depending on the ACTs administered, we divided the patients into three treatment groups: 27 treated with AP, 31 treated with DP, and 31 treated with AL. ECGs and other indicators were recorded before and after treatment. The QT interval was calculated using Fridericia’s and Bazett’s formulas.

The inclusion criteria were as follows: (1) malaria symptoms; (2) malaria falciparum found in peripheral blood smears on microscopic examination, and (3) no antimalarial medications taken 7 days before enrollment.

The exclusion criteria were as follows: (1) pregnant or breastfeeding, (2) age < 7 or > 65 years, (3) nonmalarial febrile disease, (4) taken antimalarial medicine in the past 7 days; and (4) history of allergic reactions to DP, AL, AP, or similar drugs.

### Evaluation of clinical indicators

We defined fever as a body temperature > 37.3°C. Body temperature of febrile patients was measured every 8 h until it remained normal for 24 h, and then measured once a day. Thick and thin blood smears were taken at 07:00 and 17:00 from the day of the first dose to the day of parasite clearance. Negative blood smears were defined as the examination of 200 fields, each of which had parasites less than 1/200 white blood cells. Parasite clearance was defined as three consecutive negative blood smears.

### ECG measurements

ECGs were obtained before treatment and 4 h after the final dose. All ECG recordings were sent to the First Affiliated Hospital of Guangzhou University of Chinese Medicine for blinded manual adjudication. QTcF and QTcB were corrected using Fridericia’s and Bazett’s formulas, respectively, as follows:

$$QTcF=QT/{RR}^{1/3}$$

$$QTcB=QT/{RR}^{1/2}$$

ΔQTc was defined as the difference in QTc before and after drug intervention. Moderate and severe prolongation were defined as the QT interval prolongation > 30 ms and > 60 ms, respectively^25^.

### Follow-up

At enrollment, all patients underwent comprehensive physical examinations, including assessment of clinical symptoms of malaria and neurological examinations. Microscopic examinations of thick and thin blood smears confirmed the presence of *Plasmodium* species and its corresponding load. Adverse drug reactions were monitored and recorded. If vomiting occurred within 1 hour of taking the drug, the drug was readministered. Patients were hospitalized for 7 days or until malaria symptoms disappeared or the parasites cleared. All patients were followed up weekly from the start of treatment to the 28^th^ day. If any discomfort occurred during the follow-up, patients could return to the hospital for additional follow-up. 

### Statistical analysis

After performing normality and variance homogeneity tests, the rank sum test (Stata 13.0) or chi-square test was used to count the medians or constituent ratios. *P*< 0.05 was considered significant. 

## RESULTS

### Patient recruitment and follow-up

We recruited 89 patients: 31 in the DP group, 31 in the AL group, and 27 in the AP group. Among them, 67 (75.28%) were men, 22 (24.72%) were women and 35 (39.33%) were aged ≤ 20 years, 31 (34.83%) were aged 21-30 years, 13 (14.61%) were aged 31-40 years, 7 (7.87%) were aged 41-50 years, and 3 (3.37%) were aged > 50 years. Table 1 displays the age, weight, and other baseline characteristics of the three groups.

**TABLE 1:** Baseline information of the three treatment groups.

	DP		AL		AP	
	n	%	n	%	n	%
**Gender**						
Male	25	80.65	25	80.65	17	62.96
Female	6	19.35	6	19.35	10	37.04
**Variable**	**Median**	**95% CI**	**Median**	**95% CI**	**Median**	**95% CI**
Age (years)	23	21-23	23	22-23	29	26-32
Weight (kg)	50	43.50-53	49	43.50-52.50	49	43.93-54.07
Body temperature (°C)	38.8	37.90-39.05	38.9	38.10-39.25	38.5	37.49-39.01
Fever recovery time (h)	36	26.74-42	33.5	30-42	36	26.26-38.08
*Plasmodium* removal time (h)	36	26.74-42	33.5	30-42	36	26.26-38.08

**DP:** dihydroartemisinin-piperaquine; **AL:** artemether-lumefantrine; **AP:** artemisinin-piperaquine; **CI:** confidence interval.

Comparison of the data of three groups: age (*Hc* = 0.483, *P* = 0.785); weight (*Hc* = 0.025, *P* = 0.988); *Plasmodium* density (*Hc* = 2.080, *P* = 0.353); temperature (*Hc* = 1.182, *P* = 0.554); *Plasmodium* removal time (*Hc* = 1.180, *P* = 0.554); and less recovery time (*Hc* = 0.672, *P* = 0.715). No significant difference was observed.

### Treatment effectiveness

At baseline, the DP group had 31 patients with fever, AL group had 30 patients with fever and 1 with fever but normal baseline body temperature, and AP group had 23 patients with fever and 4 with fever but normal baseline body temperature. The time of recovery from fever was not recorded for 1 patient in the AP group. Body temperatures of all febrile patients returned to normal during the 7-day hospitalization. Blood smears of all patients exhibited parasite clearance during this period. The time to parasite clearance for one and three patients in AL and AP groups, respectively, could not be calculated owing to failure of collection of blood smears at a specific time. Table 1 summarizes the data on fever and plasma load.

### ECG data analysis

**FIGURE 1:**
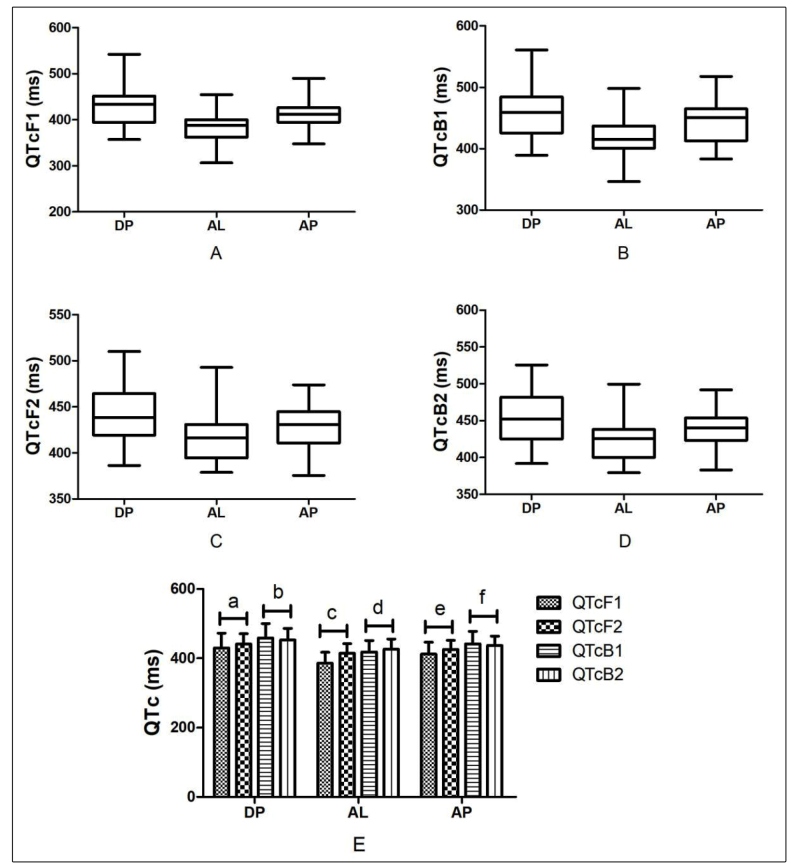
Comparison of ECG results of three treatment groups. **QTcF1:** QTcF before medication; **QTcF2:** QTcF after medication; **QTcB1:** QTcB before medication; **QTcB2:** QTcB after medication. a: Z = −1.764, P = 0.078; b: Z = 0.549, P = 0.583; c: Z = −3.214, P = 0.001; d: Z = −0.353, P = 0.724; e: Z = −1.867, P = 0.062; f: Z = 0.686, P = 0.423.

Because of the influence of fever, the difference in heart rate between patients before and after treatment is significant, necessitating the use of corrected QT. All DP, AL, and AP groups exhibited a prolonged QT interval. In the DP group, 22 patients (70.97%) had QTcF prolongation, with 7 (22.58%) and 3 (9.68%) having moderate and severe prolongations, respectively. Similarly, 14 patients (45.16%) had QTcB prolongation, with 2 (6.45%) patients each having moderate and severe prolongations. In the AL group, 24 patients (77.42%) had QTcF prolongation, with 7 (22.58%) and 5 (16.13%) having moderate and severe prolongations, respectively. Similarly, 16 patients (51.61%) had QTcB prolongation, with 2 (6.45%) and 5 (16.13%) having moderate and severe prolongations, respectively. In the AP group, 15 patients (55.56%) had QTcF prolongation, with 6 (22.22%) and 2 (7.41%) having moderate and severe prolongations, respectively. Similarly, 13 patients (48.15%) had QTcB prolongation, with 3 (11.11%) and 1 (3.70%) having moderate and severe prolongations, respectively. We did not observe drug-related QTc > 500 ms in the AL and AP groups; one case of QTcF and one case of QTcB > 500 ms in the DP group were considered to be drug-related. The comparison revealed that the incidence of QT interval prolongation among the three groups was not significantly different. We did not observe TdP or other arrhythmias in this study. Additionally, ΔQTcF and ΔQTcB values of the three groups were not significantly different. Comparing the QTc before and after the treatment, we found that QTcF in the AL group was significantly prolonged after the treatment, and there was no significant difference in QTc before and after the treatment in the other two groups. Table 2, Table 3 and Figure 1 summarize the ECG results.

**TABLE 2:** Prolongation of the QT interval among the three groups.

Variable	DP		AL		AP	
	N	%	n	%	n	%
QTcF prolongation*	22	70.97	24	77.42	15	55.56
60 ms ≥ ΔQTcF> 30 ms*	7	22.58	7	22.58	6	22.22
ΔQTcF > 60 ms*	3	9.68	5	16.13	2	7.41
QTcB prolongation*	14	45.16	16	51.61	13	48.15
60 ms ≥ ΔQTcB >30 ms*	2	6.45	2	6.45	3	11.11
ΔQTcB > 60 ms*	2	6.45	5	16.13	1	3.70

**DP:** dihydroartemisinin-piperaquine; **AL**: artemether-lumefantrine; **AP:** artemisinin-piperaquine.

QTcF = QT/RR^1/3^; QTcB = QT/RR^1/2^.

ΔQTc is defined as the difference in QTc before and after treatment.

Moderate prolongation is defined as ΔQTc > 30 ms, and severe prolongation is defined as ΔQTc > 60 ms.

*No significant difference was observed.

**TABLE 3:** QTc and ΔQTc before and after medication in each group.

Variable	Group					
	DP		AL		AP	
	Median	95% CI	Median	95% CI	Median	95% CI
QTcF1 (ms)	433.83	406.05-446.44	388.06	375.73-395.56	412.31	394.87-424.02
QTcF2 (ms)	438.57	428.38-458.75	416.49	400.56-421.60	430.89	416.76-440.15
ΔQTcF (ms)	13.24	2.82-29.17	21.61	9.68-34.61	5.8	−10.57-26.70
QTcB1 (ms)	458.98	436.18-479.13	415.33	403.32-427.33	450.6	417.13-460.21
QTcB2 (ms)	458.98	436.18-479.13	415.33	403.32-427.33	450.6	417.13-460.21
ΔQTcB (ms)	−3.5	−15.51-9.94	0.97	−11.83-9.82	0	−28.44-12.70

**DP:** dihydroartemisinin-piperaquine; **AL**: artemether-lumefantrine; **AP:** artemisinin-piperaquine; **CI:** confidence interval.

**QTcF1:** QTcF before medication; **QTcF2:** QTcF after medication; **QTcB1:** QTcB before medication; **QTcB2:** QTcB after medication.

QTcF1 > 500 ms: DP, 2 cases; AL, 0 cases; AP, 0 cases.

QTcF2 > 500 ms: DP, 1 case; AL, 0 cases; AP, 0 cases.

QTcB1 > 500 ms: DP, 3 cases; AL, 0 cases; AP, 2 cases.

QTcB2 > 500 ms: DP, 2 cases; AL, 0 cases; AP, 0 cases.

Comparing the ΔQTcF of the three groups, *Hc*= 2.154, *P*= 0.341; comparing the ΔQTcB of the three groups *Hc*= 0.753, *P*= 0.686. No significant difference was observed.

## DISCUSSION

Malaria is an infectious disease caused by *Plasmodium* species and transmitted from humans to humans or from animals to humans by mosquitoes. The WHO guidelines^17^ recommend using RDT or microscopy to confirm the diagnosis of malaria in suspected cases. Anopheles mosquitoes are the most important vectors for the spread of malaria. There are no specific signs or symptoms of malaria, except for the characteristic fever. Understanding the emerging patterns of the malaria epidemic can increase the availability of treatments and other control actions^26^. The overall trend in the incidence of malaria has decreased. However, according to the 2019 World Malaria Report, there were 228 million malaria cases worldwide in 2018. Malaria still greatly influences public health^27^. We thus require safe, effective, and widely available antimalarials.

The WHO Malaria Policy Advisory Committee (MPAC), which met on March 22-24, 2017, expressed that DP may account for 1 cardiac death among 200,000 treated patients. However, AL was not associated with such sudden deaths. The MPAC mentioned that the cardiotoxicity of halofantrine, in addition to other antimalarial drugs, is negligible but not investigated in detail. Cases of TdP or life-threatening arrhythmias caused by drugs are rare, but there is currently no simple screening method to identify high-risk individuals.

The mechanism of cardiac ventricular myocyte repolarization is mainly related to the outward current of potassium ions (I_k_), especially those affected by the human fibroblast subunit encoded on chromosome 7 (ether-a-go-go-related gene, hERG)^28^. Mutation of hERG can cause long QT syndrome, which may lead to TdP or even sudden death^29^ ^,^ ^30^. Drugs that cause TdP block hERG cardiac potassium channels, but not all drugs that block hERG cause TdP^31^. The U.S. Food and Drug Administration noted that the QTc interval prolongation > 60 ms increases the risk of TdP. When the voltage-gated channels are opened, the drug binds to the channel, causing inhibition of the channel and prolongation of the QT interval^20^. Prolongation of the QT interval indicates delayed cardiac repolarization that increases the risk of arrhythmia, such as TdP, and even leads to sudden cardiac death^32^ ^-^ ^34^. Factors that increase the risk of TdP include QTc prolongation, sex (female), advanced age (> 65 years), bradycardia, hypokalemia, and underlying heart disease. Although many drugs may cause TdP, the reported cases have at least one or more of the above factors. Antimalarial drug safety assessments include antimalarial drug-related QTc prolongation^21^ ^,^ ^22^. *P. falciparum* can be isolated from cardiac microvessels, though severe malaria rarely causes significant myocardial dysfunction or arrhythmia; therefore, we can exclude the impact of *Plasmodium* species on cardiac activity^35^. Most patients with malaria were febrile. The comparison of QT intervals in malaria cases needs to be corrected because for every 1°C increase in body temperature, the heart rate increases by 8.5 bpm^20^.

Owing to the influence of sex hormones, the QT interval begins to show sex-based differences during puberty; the QT intervals of men gradually become longer than those of women^36^. Female sex is a risk factor for QT prolongation. There was a sex bias in this study with 75.28% men and 24.72% women. Simultaneously, we excluded patients with a history of heart disease from the study to avoid any serious events. Thus, we cannot extrapolate the results of this study to all malaria patients, especially those with a history of heart disease. Many risk factors still need to be considered when patients experience QT prolongation after receiving DP, AL, or AP compounds. The relationship between QT prolongation and sudden death is complicated, but QT prolongation significantly increases the risk of TdP^37^. Large sample studies on healthy subjects and malaria patients are required to conclusively comment on the cardiac safety of these three drugs.

In people with low immunity or severe malaria, higher levels of proinflammatory cytokines (e.g., interleukin-6) during acute infection may also affect the QT interval by inhibiting the function of cardiomyocyte ion channels^38^ ^,^ ^39^. *Plasmodium* infection can cause multiple organ dysfunctions, but its impact on the heart is relatively small. Arrhythmia caused by malaria is rare. Although *Plasmodium* can be isolated from cardiomyocytes in severe cases of malaria, heart function can be maintained at normal levels^40^. Artemisinin has a short half-life; thus, drugs compatible with ACT are the main factors leading to QT interval prolongation, which is related to the long-term efficacy of the compound^41^ ^,^ ^42^. We did not find any clinical manifestation indicating or ECG features of arrhythmia; hence, the treatment-induced QT interval prolongation in some patients in this study may not be clinically significant^43^. This indicates that the use of these three ACTs at the clinically recommended dose does not increase the risk of TdP or other arrhythmias.

In this study, we retrospectively studied the effects of three artemisinin complexes on the ECG findings of malaria patients by conducting a case-series analysis. This study has some limitations. First, there was a certain bias with respect to the study subjects. To avoid serious adverse events, only patients with non-severe malaria were included in the study. Because local men have more exposure to the field labor environment than women, there were more cases of malaria in men; hence, there is a sex bias among the cases in this study. Second, because of the medical conditions at that time, the ion changes before and after treatment were not monitored, making it impossible for us to evaluate the effect of the drugs on the QT interval through ion channels. Third, the ECGs of patients with prolonged QT intervals were not continuously tracked; thus, we cannot evaluate the prognostic ability of patients’ ECGs.

Though DP, AL, and AP treatment can cause QT prolongation in some malaria patients, we did not observe TdP or other types of arrhythmias in this study. Therefore, we can consider that the use of these three ACTs at their clinically recommended doses does not produce significant QT interval prolongation and that they can be safely used for malaria treatment. However, this study did not represent all patients with malaria. Patients with a history of heart disease or factors that may cause QT interval prolongation should be closely monitored during medication.
